# Longitudinal changes in infrapatellar fat pad fibrosis following anterior cruciate ligament reconstruction: a retrospective cohort study

**DOI:** 10.1186/s43019-026-00345-1

**Published:** 2026-11-13

**Authors:** Shunji Takei, Yoshiyuki Imoo, Hiroto Onuma, Takeo Mammoto, Hiroyuki Kobayashi

**Affiliations:** 1 Department of Rehabilitation, Tsukuba University Hospital Mito Clinical Education and Training Center, Mito Kyodo General Hospital, Mito, Japan; 2 Doctoral Program in Medical Sciences, Graduate School of Comprehensive Human Sciences, University of Tsukuba, Tsukuba, Japan; 3 Department of Orthopedic Surgery, Tsukuba University Hospital Mito Clinical Education and Training Center, Mito Kyodo General Hospital, Mito, Japan; 4 Department of Internal Medicine, Tsukuba University Hospital Mito Clinical Education and Training Center, Mito Kyodo General Hospital, Mito, Japan

**Keywords:** anterior cruciate ligament reconstruction, infrapatellar fat pad, fibrosis, magnetic resonance imaging, longitudinal study

## Abstract

**Background:**

Infrapatellar fat pad (IFP) fibrosis is implicated in anterior knee pain, limited range of motion (ROM), and posttraumatic osteoarthritis (PTOA) after anterior cruciate ligament reconstruction (ACLR). However, its precise longitudinal progression remains unclear. This study aimed to elucidate the dynamic changes in IFP fibrosis from preoperative to 12 months post-ACLR using magnetic resonance imaging (MRI).

**Methods:**

We retrospectively analyzed consecutive patients who underwent anatomic double-bundle ACLR using autologous hamstring tendon grafts. IFP fibrosis was semiquantitatively graded (grades 0–5) using proton density-weighted (PDW) sagittal MR images obtained preoperatively and at 6 and 12 months postoperatively. Patients were categorized into mild (grades 0–3) and severe (grades 4–5) IFP fibrosis groups. Temporal changes in the proportion of the severe group across the three time points were evaluated using Cochran’s *Q* test, followed by the Bonferroni-corrected McNemar test for pairwise comparisons.

**Results:**

Of 558 consecutive cases screened, 169 patients (mean age, 23.9 years) were included in the final analysis. The proportion of patients classified into the severe fibrosis group significantly changed over time (Cochran’s *Q* test, *χ*^2^ (2) = 24.0, *p* < 0.001). Compared with the preoperative proportion (1.2%), the proportion of patients in the severe group increased significantly at 6 months postoperatively (8.3%, *p* = 0.003) and persisted at 12 months (8.3%, *p* = 0.003). Crucially, 91.1% of the patients showed no change in fibrosis grade between 6 and 12 months. This demonstrates that IFP fibrosis significantly progressed within the first 6 months post-ACLR, with minimal subsequent changes up to 12 months.

**Conclusions:**

IFP fibrosis is an early and persistent pathological event following ACLR and is established primarily within the first 6 postoperative months. This early onset suggests a critical time window for targeted interventions to mitigate inflammation and prevent the irreversible establishment of IFP fibrosis, providing a basis to investigate the long-term PTOA risk.

## Background

Anterior cruciate ligament (ACL) injuries represent a major public health concern, particularly among active populations such as young individuals and sports enthusiasts [1, 2]. Although ACL reconstruction (ACLR) aims to restore knee stability and function [3], it often fails to prevent the long-term development of posttraumatic osteoarthritis (PTOA) [4, 5]. This progression suggests the involvement of pathological mechanisms beyond mechanical instability, such as traumatic damage to the menisci or cartilage at the time of injury and subsequent kinematic changes [6–8]. Additionally, persistent intraarticular inflammation, characterized by a dysregulated inflammatory response and elevated proinflammatory cytokines, is known to promote cartilage degradation [9, 10]. The infrapatellar fat pad (IFP) is a vital intraarticular organ that secretes cytokines and plays a pivotal role in joint homeostasis and disease progression [11–13]. Pathological changes in the IFP, particularly fibrosis, are known contributors to postoperative complications such as restricted range of motion (ROM), anterior knee pain, and risk of future cartilage damage and PTOA [14–16].

Fibrosis is a hallmark of pathological changes in the IFP, characterized by chronic inflammation, myofibroblast differentiation, and excessive collagen deposition driven by an acute intraarticular inflammatory response following ACLR [17–19]. This pathological process is a key target of preventive interventions. Magnetic resonance imaging (MRI) is a reliable and noninvasive tool for the semiquantitative assessment of fibrotic changes [20–22]. However, existing literature on IFP fibrosis following ACLR predominantly relies on single time points or cross-sectional evaluations [19, 21]. Consequently, the precise timing and dynamic longitudinal course of IFP fibrosis progression, particularly during the critical period from preoperative to 1 year postoperatively, remain unknown.

Understanding the dynamic course of IFP pathology is essential to identify the critical time window for targeted interventions before fibrosis becomes irreversible. To our knowledge, this is the first longitudinal report documenting the dynamic changes in IFP fibrosis post-ACLR using serial MRI evaluations from the preoperative period to 12 months postoperatively. The primary objective of this study was to elucidate the longitudinal changes in IFP fibrosis following ACLR based on serial MRI evaluations at preoperative, 6-month, and 12-month postoperative time points. By clarifying the timing of the onset and progression of IFP fibrosis, this study aimed to provide valuable insights into the early pathological events contributing to chronic knee complications post-ACLR and to support the development of strategies for preventing PTOA.

## Methods

### Patients

This retrospective cohort study included consecutive patients who underwent primary anatomic double-bundle ACLR surgery at our institution between 2012 and 2024. The inclusion criteria were the availability of complete MRI datasets at three specific time points: preoperatively, and at 6 and 12 months postoperatively. Patients were excluded based on the following criteria: history of revision ACLR surgery, prior ipsilateral knee injury or surgical interventions, use of bone-patellar tendon-bone (BTB) autografts, and occurrence of postoperative joint infections. Additionally, patients whose preoperative MRI had been performed at other institutions, or those with missing or incomplete MRI datasets at any of the designated follow-up time points, were also excluded from the final analysis.

### Surgical procedure

All patients underwent arthroscopic anatomic double-bundle ACL reconstruction with remnant preservation using autologous hamstring tendons. Standard arthroscopy through the anterolateral and anteromedial portals confirmed ACL rupture, and the menisci and cartilage were evaluated. Articular cartilage status was graded according to the Outerbridge classification, with grade 2 or higher defined as cartilage damage. The mucosal ligament was preserved, and the IFP or synovium around the portals was minimally resected only when visualization was impaired. The grafts consisted of either the semitendinosus tendon alone (*n* = 156) or in combination with the gracilis tendon (*n* = 13). The femoral and tibial tunnels were created at the anatomical attachment sites of the anteromedial and posterolateral bundles using the outside-in technique for femoral drilling. Femoral fixation was achieved using a suspensory device (EndoButton CL, Smith & Nephew; or PermaLOOP, DePuy Synthes), and tibial fixation was performed using a double-spike plate (Smith & Nephew).

### Postoperative rehabilitation

All patients followed a standardized postoperative rehabilitation protocol. Nonweight-bearing was maintained during the first postoperative week, followed by 50% partial weight-bearing initiated in the second week, and progression to full weight-bearing from the third week. An ACL functional brace was worn for approximately 3 months postoperatively; the timing of its discontinuation was determined by the attending surgeon based on clinical findings and MRI evaluation. Return to sports was generally permitted after 6 months, contingent upon sufficient recovery of muscle strength and physical function.

### MRI acquisition and IFP fibrosis assessment

**Fig. 1:**
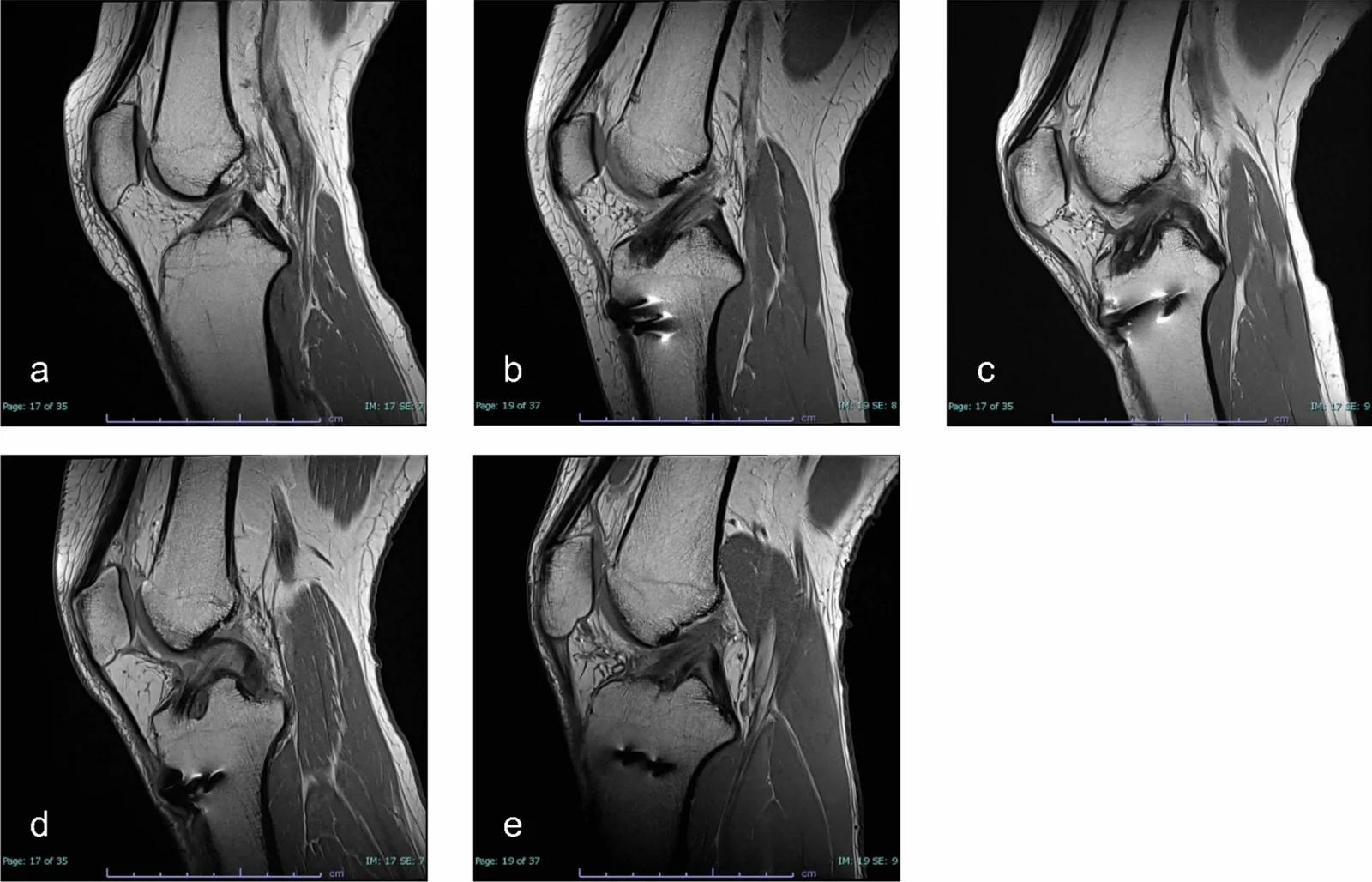
Representative sagittal proton density-weighted MR images illustrating the grading system for infrapatellar fat pad (IFP) fibrosis. **a** Grade 0: no fibrosis, **b** grade 1: focal fibrosis, **c** grade 2: incomplete band fibrosis, **d** grade 3: complete band fibrosis, and **e** grade 4: infiltrated fibrosis. Fibrotic changes are identified as regions of low signal intensity compared with the surrounding hyperintense adipose tissue. No cases of grade 5 (diffuse and infiltrated fibrosis) were observed in this study cohort

Longitudinal changes in IFP fibrosis were evaluated using proton density-weighted (PDW) sagittal images acquired via 1.5 T or 3.0 T MRI at three standardized time points: preoperatively, 6 months postoperatively, and 12 months postoperatively. Fibrosis was graded using the semiquantitative criteria proposed by Nakagawa et al. [21], which scored fibrosis from grade 0 (none) to grade 5 (diffuse and infiltrated fibrosis). Representative images of this grading system are shown in Fig. 1.

The evaluation of IFP fibrosis was conducted by two blinded investigators experienced in orthopedic imaging, without access to patient information or surgical details. To ensure evaluation reliability, these two independent, blinded investigators assessed all cases. The inter-rater intraclass correlation coefficient (ICC) was 0.841, indicating good reliability, and the intra-rater ICC of the primary investigator was 0.961, indicating excellent reliability.

### Clinical assessment

To examine the clinical relevance of IFP fibrosis, clinical outcomes were evaluated using the Japanese Orthopaedic Association (JOA) score at 6 and 12 months postoperatively. The JOA score for knee diseases comprises four subitems: pain on walking, pain on climbing stairs, ROM, and joint effusion, with a maximum possible score of 100 points [23].

### Statistical analysis

The patients were classified into two groups based on the criteria established by Nakagawa et al. [21]: mild IFP fibrosis (grade 0–3) and severe IFP fibrosis (grade 4–5). Multivariate analysis was not performed due to the limited number of cases in the severe fibrosis group (*n* = 14), which precluded reliable statistical estimation. Continuous variables were compared between the two groups using the Student’s *t*-test or Mann–Whitney *U* test, as appropriate, and categorical variables were compared using the chi-square test or Fisher’s exact test. Statistical analyses were performed using R software (ver. 4.5.0). Cochran’s *Q* test for repeated measures on dichotomous data was used to evaluate changes in the proportion of the severe group across the three time points (preoperatively, 6 months, and 12 months postoperatively). When significant differences were observed, comparisons between specific time points were performed using the Bonferroni-corrected McNemar test for paired data. The level of significance was set at *p* < 0.05. To assess the adequacy of the sample size for our primary outcome, a post hoc power analysis was performed using G*Power (version 3.1.9.7). For the comparison between the preoperative and 6-month postoperative stages (McNemar test), the calculated statistical power (1-β) was 0.75 (with an alpha of 0.05, a total sample size of 169, and a proportion of discordant pairs of 0.082).

## Results

### Patient characteristics

A total of 558 consecutive patients who underwent ACLR during the study period were initially screened. After applying exclusion criteria—including revision surgery, prior knee injury or surgery, use of BTB autografts, and postoperative infection—513 patients remained eligible. Of these, 160 patients were excluded because their preoperative MRI was performed at other institutions, and an additional 184 patients were excluded due to missing MRI data at one or more follow-up time points. The primary reasons for missing data were administrative or logistical factors, such as relocation or follow-up at other clinics, rather than clinical complications or patient refusal due to poor outcomes. Ultimately, 169 patients met all criteria and were included in the analysis. The study population consisted of 61 males and 108 females, with a mean age of 23.9 ± 11.9 years. The demographic characteristics are summarized in Table 1. Baseline comparisons between the mild and severe fibrosis groups revealed no significant differences in age, body mass index (BMI), operative time, cartilage injury, or meniscus treatment. However, the severe fibrosis group had a significantly higher proportion of males (*p* = 0.038) and greater mean height (*p* = 0.016) compared with the mild group. Additionally, a significant difference was observed in the distribution of preoperative IFP fibrosis grades (*p* = 0.027). As stated in the Methods, the limited number of patients in the severe fibrosis group (*n* = 14) precluded multivariate analysis. MRI was performed using either 1.5 T or 3.0 T scanners throughout the study period (1.5 T: *n* = 24; 3.0 T: *n* = 145).

**Table 1:** Patient characteristics

Characteristics	All (*n* = 169)	Comparative groups		
		Mild group (*n* = 155)	Severe group (*n* = 14)	*p*-value
Age (years)	23.9 ± 11.9	24.2 ± 12.0	21.5 ± 10.9	0.655
Sex, male/female, *n*	61/108	52/103	9/5	0.038*
Affected side, right/left, *n*	77/92	69/86	8/6	0.364
Height (cm)	163.8 ± 8.8	163.3 ± 8.8	169.1 ± 7.3	0.016*
Body weight (kg)	63.2 ± 12.5	62.8 ± 12.7	67.2 ± 9.0	0.071
BMI	23.4 ± 3.5	23.4 ± 3.7	23.4 ± 2.1	0.492
Operation time (min)	114.3 ± 26.4	113.3 ± 25.6	125.1 ± 32.5	0.120
Cartilage injury				1.000
Present	44 (26.0%)	41 (26.5%)	3 (21.4%)	
Absent	125 (74.0%)	114 (73.5%)	11 (78.6%)	
Meniscus injury				0.052
Present	125 (74.0%)	118 (76.1%)	7 (50.0%)	
Absent	44 (26.0%)	37 (23.9%)	7 (50.0%)	
Meniscus treatment				0.430
Untreated	89 (52.7%)	79 (51.0%)	10 (71.4%)	
Sutured	45 (26.6%)	43 (27.7%)	2 (14.3%)	
Excised	35 (20.7%)	33 (21.3%)	2 (14.3%)	
Preoperative IFP fibrosis grade				0.027*
Grade 0	16 (9.5%)	15 (9.7%)	1 (7.1%)	
Grade 1	48 (28.4%)	44 (28.4%)	4 (28.6%)	
Grade 2	97 (57.4%)	90 (58.1%)	7 (50.0%)	
Grade 3	6 (3.6%)	6 (3.9%)	0 (0.0%)	
Grade 4	2 (1.2%)	0 (0.0%)	2 (14.3%)	
Grade 5	0 (0.0%)	0 (0.0%)	0 (0.0%)	
JOA knee score				
6 Months postoperatively				
Total score	99.2 ± 2.2	99.3 ± 1.8	97.9 ± 4.7	0.287
Pain on walking	30.0 ± 0.4	30.0 ± 0.0	29.6 ± 1.3	< 0.001*
Pain on stairs	25.1 ± 1.4	25.1 ± 1.3	24.3 ± 2.7	0.169
Range of motion	34.3 ± 1.8	34.3 ± 1.8	33.9 ± 2.1	0.470
Joint effusion	9.9 ± 0.7	9.9 ± 0.7	10.0 ± 0.0	0.609
12 months postoperatively				
Total score	99.8 ± 1.2	99.7 ± 1.2	100.0 ± 0.0	0.423
Pain on walking	30.0 ± 0.0	30.0 ± 0.0	30.0 ± 0.0	NA
Pain on stairs	25.2 ± 1.1	25.2 ± 1.0	25.4 ± 1.3	0.664
Range of motion	34.6 ± 1.4	34.6 ± 1.4	34.6 ± 1.3	0.877
Joint effusion	9.9 ± 0.5	9.9 ± 0.6	10.0 ± 0.0	0.681

Continuous variables are presented as mean ± standard deviation (SD); categorical variables are presented as *n* (%)

*BMI* body mass index, *SD* standard deviation, *JOA score* Japanese Orthopaedic Association score, *NA* not applicable

Statistical significance was determined using the Student’s *t*-test, Mann–Whitney *U* test, chi-square test, or Fisher’s exact test, as appropriate. *p < 0.05

Regarding clinical outcomes, the severe fibrosis group showed a significantly lower JOA subscore for “pain on walking” at 6 months postoperatively compared with the mild fibrosis group (*p* < 0.001). However, no significant differences were observed in other subscores, such as ROM or pain on climbing stairs, at either 6 or 12 months postoperatively.

### Longitudinal changes in fibrosis grade distribution

The results of this study demonstrated a clear longitudinal pattern in the progression of IFP fibrosis after ACLR. IFP fibrosis progressed for up to 6 months postoperatively and showed little change thereafter.

Table 2 shows the distribution of the IFP fibrosis grades at the three time points. Preoperatively, most patients (95.3%) exhibited grade 2 or lower fibrosis, indicating a mild-to-minimal pathological state.

**Table 2:** Distribution of infrapatellar fat pad (IFP) fibrosis grades over time

IFP fibrosis grade	Preoperatively, *n* (%)	6 Months postoperatively, *n* (%)	12 Months postoperatively, *n* (%)
Grade 0	16 (9.5%)	3 (1.8%)	1 (0.6%)
Grade 1	48 (28.4%)	9 (5.3%)	7 (4.1%)
Grade 2	97 (57.4%)	99 (58.6%)	93 (55.0%)
Grade 3	6 (3.6%)	44 (26.0%)	54 (32.0%)
Grade 4	2 (1.2%)	14 (8.3%)	14 (8.3%)
Grade 5	0 (0.0%)	0 (0.0%)	0 (0.0%)

Fibrosis progression was observed within the first 6 months postoperatively. By 6 months, the proportion of patients with grade 3 or higher fibrosis had increased sharply to 34.3% (58/169).

Between 6 and 12 months postoperatively, the changes in fibrosis severity were minimal, indicating a plateau in the pathological process. During this period, 91.1% (154/169) of patients showed no change in their assigned fibrosis grade. At 12 months, 40.2% of the patients (68/169) exhibited grade 3 or higher fibrosis, confirming that the fibrosis established at 6 months largely persisted throughout the 12-month follow-up.

### Comparison of severe fibrosis proportions

Table 3 shows the proportion of patients with severe fibrosis (grade 4–5) over time. Comparison across the three time points using Cochran’s *Q* test revealed a significant difference in the proportion of the severe group (*χ*^2^ (2) = 24.0, *p* < 0.001).

**Table 3:** Comparison of mild and severe infrapatellar fat pad (IFP) fibrosis groups

	Preoperatively, *n* (%)	6 Months postoperatively, *n* (%)	12 Months postoperatively, *n* (%)
Mild fibrosis (grade 0–3)	167 (98.8%)	155 (91.7%)	155 (91.7%)
Severe fibrosis (grade 4–5)	2 (1.2%)	14 (8.3%)	14 (8.3%)

Pairwise comparisons adjusted using Bonferroni correction showed a significant increase in the proportion of patients in the severe group at 6 months postoperatively (8.3%) compared with preoperatively (1.2%) (*p* = 0.003). Similarly, a significant increase was observed at 12 months postoperatively (8.3%) compared with preoperatively (1.2%) (*p* = 0.003).

The McNemar test was not conducted for comparison between 6 and 12 months postoperatively because there were no discordant data (i.e., no change in severe/mild status). This finding suggests that the increase in severe cases occurs within the first 6 months post-ACLR and remains stable thereafter up to 12 months.

## Discussion

This study addresses a critical gap in the literature by elucidating a distinct and persistent pattern: IFP fibrosis progresses significantly within the first 6 months postsurgery and subsequently stabilizes up to the 12-month follow-up. Given that IFP fibrosis is a recognized contributor to anterior knee pain, ROM limitations, and long-term risk of PTOA [14–16], clarifying the precise timing of its onset and progression is crucial. This insight contributes to our understanding of the critical time window before fibrosis becomes irreversible, which may provide a conceptual framework for future studies exploring the optimal timing for prophylactic clinical intervention. Previous studies, limited to cross-sectional evaluations, could only hypothesize the speed of this process. Therefore, our findings are the first to quantitatively clarify the rapid, early-stage progression of IFP fibrosis, thereby providing significant academic and clinical value.

Our longitudinal findings demonstrated that the most significant progression of IFP fibrosis occurred within the first 6 months post-ACLR, with minimal subsequent changes thereafter. The sharp increase in grade 3 or higher fibrosis at 6 months postoperatively, affecting over one-third of the cohort, suggests that pathological changes may become established relatively early in the postoperative course and persist thereafter. This persistent plateau from 6 to 12 months stands in contrast to the historical report by Tang et al., who observed that fibrous scars peaked at 6 months but disappeared in nearly half of the cases by 1 year. Several systematic factors may explain this discrepancy. Modern anatomic double-bundle ACLR with remnant preservation, as performed in our cohort, creates a distinct biological environment compared with the historical techniques utilized by Tang et al. The preservation of native tissue and more extensive bone tunnel creation may contribute to a different intraarticular inflammatory profile, potentially stabilizing the fibrotic response. Furthermore, the 6-point grading system by Nakagawa et al. provides higher resolution than the 3-point score used in the previous study, enabling the detection of subtle or diffuse infiltration that might have otherwise been categorized as “disappeared.” Although grade 3 represents a structurally continuous fibrotic band, we classified it within the mild fibrosis group in accordance with the original criteria proposed by Nakagawa et al. to maintain consistency with previous literature. However, this classification may underestimate clinically relevant structural changes, and alternative thresholds should be explored in future studies. Crucially, unlike the cross-sectional analysis with inconsistent timings (ranging from 3 to >18 months) in the previous report, our longitudinal design with standardized MRI time points for all 169 patients minimizes temporal bias. These methodological strengths suggest that IFP fibrosis, once established early postoperatively, typically reaches a plateau rather than regressing as previously suggested. This rapid onset of fibrosis is closely linked to the acute and subacute inflammatory responses following ACL injury and subsequent surgery. Tissue injury triggers a cascade in which platelets and immune cells aggregate, releasing potent profibrotic growth factors such as transforming growth factor beta (TGF-β) and platelet-derived growth factor (PDGF). These factors drive the differentiation of resident fibroblasts into myofibroblasts, leading to excessive and persistent collagen production, which forms stiff fibrotic tissue [24]. Previous studies have reported peaks in inflammatory cytokine levels several weeks to months postoperatively, which coincide with the observation of fibrotic findings on MRI [21, 25]. Our findings, based on objective longitudinal MRI data, corroborated this molecular pathological timeline at the macroscopic level.

The fact that 8.3% of the patients exhibited severe fibrosis (grade 4–5) at 6 months postsurgery, with no change in severity at 12 months, suggests that IFP fibrosis, once established early, is highly resistant to natural regression. Baseline comparisons (Table 1) showed a higher proportion of males (*p* = 0.038), greater height (*p* = 0.016), and a different distribution of preoperative IFP fibrosis grades (*p* = 0.027) in the severe fibrosis group. Although only two patients had preoperative grade 4 fibrosis, both developed severe fibrosis at 6 months, suggesting that advanced preoperative IFP fibrosis may be associated with an increased risk of severe postoperative fibrosis and may have implications for preoperative risk stratification. However, because the severe group was small (*n* = 14), only two patients had preoperative grade 4 fibrosis, and multivariable adjustment was not feasible, the independent contributions of preoperative fibrosis grade, sex, and height could not be determined. These associations should be confirmed in larger multicenter studies. Further molecular characterization of the fibroinflammatory environment, particularly the role of TGF-β signaling, remains important for elucidating the mechanisms of persistent IFP fibrosis after ACLR. These findings further emphasize the importance of identifying strategies to prevent excessive fibrotic remodeling during the early postoperative period.

The identification of this early fibrotic window has important implications for postoperative management. Early postoperative rehabilitation emphasizing restoration of full knee extension and progressive range-of-motion exercises represents the most clinically established strategy for reducing postoperative motion loss and intraarticular adhesions after ACLR [26]. Although direct evidence that early range-of-motion exercises prevent IFP fibrosis is limited, minimizing anterior compartment adhesions may reduce subsequent fibrotic remodeling of the IFP. Rehabilitation intensity should nevertheless be individualized to avoid excessive loading of the healing graft [27]. Experimental inhibition of TGF-β signaling has also been reported to reduce postoperative arthrofibrosis [28]. However, because TGF-β contributes to normal tissue repair and graft remodeling, the efficacy and safety of this approach after ACLR remain uncertain. Further studies of localized and temporally controlled antifibrotic interventions are warranted.

Although this study provides novel insights into the longitudinal changes in IFP fibrosis post-ACLR, certain limitations must be acknowledged. First, the retrospective design of the study may have introduced variability in imaging conditions. Specifically, both 1.5 T and 3.0 T MRI scanners were utilized. The higher signal-to-noise ratio (SNR) of 3.0 T MRI might enhance the sensitivity for detecting fine, low-signal intensity fibrotic regions compared with 1.5 T MRI. Although both 1.5 T and 3.0 T MRI scanners were used in this study, the heterogeneity of field strengths could potentially introduce systematic bias in the semiquantitative grading. Future studies should ideally employ a single magnetic resonance field strength or utilize more objective, quantitative methods to minimize such potential bias. Second, the evaluation method adopts a semiquantitative grading classification [21]. While we attempted to ensure objectivity through rigorous blinding and confirmed favorable inter-rater reliability (ICC > 0.8), some degree of subjectivity in MRI interpretation is inherent to semiquantitative scoring. Furthermore, to maintain comparability with previous studies [19, 21], we defined grade 4 or higher as “severe” fibrosis. However, grade 3 (“complete band fibrosis”) represents a distinct structural change and may warrant further investigation. Although quantitative MRI parameters such as proton density fat fraction (PDFF) have been shown to reflect histopathological IFP fibrosis [22], our study relied on semiquantitative grading without histological correlation. The use of objective quantitative indices such as PDFF in future studies may help minimize potential bias associated with differences in magnetic field strength and further clarify the clinical significance of specific fibrosis grades. Third, the post hoc statistical power for the severe fibrosis group was 0.75. Although this is slightly below the conventional 0.80 threshold, it demonstrates substantial sensitivity for identifying longitudinal trends in this single-center cohort. However, the limited absolute number of patients in the severe group (*n* = 14) precluded robust multivariate adjustments for potential confounders, such as BMI or operative time. Consequently, our findings should be interpreted within these statistical constraints. While we utilized a consecutive series of patients to ensure a representative sample, larger multicenter studies are warranted to further validate these trends with greater statistical precision. Fourth, this study did not examine the direct relationship between fibrosis and clinical findings. While we tracked the pathological progression of IFP fibrosis, we did not directly investigate how this fibrosis correlated with postoperative pain, ROM limitation, or future PTOA development. Furthermore, the 12-month follow-up period in this study is likely insufficient to capture the slow, long-term progression of PTOA. Future research is essential to analyze the relationship between objective MRI findings and clinical outcomes, including patient-reported outcome measures (PROMs) and objective functional measures. Fifth, the exclusion of patients with missing MRI data may have introduced selection bias. It is possible that patients with poor clinical recovery or persistent knee stiffness were less likely to complete the follow-up imaging protocol. However, the predominant reasons for data attrition in this cohort were geographical or administrative (e.g., relocation or transfer of care), and cases where imaging was avoided due to poor clinical status were extremely limited. Therefore, we believe that the 169 patients included in this study are sufficiently representative of the standard postoperative course following ACLR. Sixth, the 12-month follow-up period is insufficient to evaluate the clinical onset of PTOA, which typically develops over years or decades post-ACLR. While our study clarifies the early dynamic changes of IFP fibrosis, long-term longitudinal studies are required to determine whether these early fibrotic changes serve as a direct predictor of future degenerative joint disease. Furthermore, PTOA is a multifactorial process significantly influenced by baseline meniscal and cartilage status. Although we considered multivariate analysis to control for these confounders, the limited number of patients in the severe fibrosis group (*n* = 14) precluded robust statistical modeling and increased the risk of overfitting. Therefore, IFP fibrosis should be interpreted as one of several potential contributors to the complex pathogenesis of PTOA. Finally, clinical evaluation using the JOA score was limited by the ceiling effect of this scoring system and the small size of the severe group. Specifically, the “pain on walking” subscore reached 30.0 ± 0.0 in all patients at 12 months postoperatively, precluding between-group comparison, while the mild fibrosis group had already reached this ceiling at 6 months. The JOA score, which was not specifically developed as a patient-reported outcome measure for young patients after ACLR, may, therefore, be insufficiently sensitive to detect subtle differences in this relatively young, high-functioning cohort. Future studies should incorporate widely used, internationally validated PROMs, such as the International Knee Documentation Committee subjective knee score or the Knee injury and Osteoarthritis Outcome Score, to assess subtle differences in symptoms and function.

## Conclusions

This study is the first longitudinal study documenting the dynamic changes in IFP fibrosis post-ACLR. Our findings demonstrate a clear pattern in which IFP fibrosis progresses significantly within the first 6 months postoperatively and persists until the 12-month follow-up. This finding suggests that the early postoperative stage is a critical window that determines the extent and persistence of IFP fibrosis, suggesting a potential link to the long-term risk of developing PTOA, which warrants further clinical investigation. Future research should focus on targeted early-phase interventions, such as controlled rehabilitation protocols and local antifibrotic strategies, to mitigate inflammation and prevent the irreversible establishment of IFP fibrosis while preserving graft healing.

## Data Availability

The datasets generated or analyzed during this study are available from the corresponding author upon reasonable request.

## Abbreviations

ACL: anterior cruciate ligament
ACLR: anterior cruciate ligament reconstruction
BMI: body mass index
BTB: bone-patellar tendon-bone
ICC: intraclass correlation coefficient
IFP: infrapatellar fat pad
JOA: Japanese Orthopaedic Association
MRI: magnetic resonance imaging
NA: not applicable
PDFF: proton density fat fraction
PDGF: platelet-derived growth factor
PDW: proton density-weighted
PROMs: patient-reported outcome measures
PTOA: post-traumatic osteoarthritis
ROM: range of motion
SD: standard deviation
SNR: signal-to-noise ratio
TGF-β: transforming growth factor-beta
